# Microtubule-Associated Proteins in Mesial Temporal Lobe Epilepsy with and without Psychiatric Comorbidities and Their Relation with Granular Cell Layer Dispersion

**DOI:** 10.1155/2013/960126

**Published:** 2013-08-27

**Authors:** Ludmyla Kandratavicius, Mariana Raquel Monteiro, Jaime Eduardo Hallak, Carlos Gilberto Carlotti, Joao Alberto Assirati, Joao Pereira Leite

**Affiliations:** ^1^Ribeirao Preto Medical School, Department of Neurosciences and Behavior, University of Sao Paulo (USP), Avenida Bandeirantes 3900, 14049-900 Ribeirao Preto, SP, Brazil; ^2^Center for Interdisciplinary Research on Applied Neurosciences (NAPNA), USP, Avenida Bandeirantes 3900, 14049-900 Ribeirao Preto, SP, Brazil; ^3^National Institute of Science and Technology in Translational Medicine (INCT-TM/CNPq), Avenida Bandeirantes 3900, 14049-900 Ribeirao Preto, SP, Brazil; ^4^Ribeirao Preto Medical School, Department of Surgery, USP, Avenida Bandeirantes 3900, 14049-900 Ribeirao Preto, SP, Brazil

## Abstract

*Background*. Despite strong association between epilepsy and psychiatric comorbidities, biological substrates are unknown. We have previously reported decreased mossy fiber sprouting in mesial temporal lobe epilepsy (MTLE) patients with psychosis and increased in those with major depression. Microtubule associated proteins (MAPs) are essentially involved in dendritic and synaptic sprouting. *Methods*. MTLE hippocampi of subjects without psychiatric history, MTLE + major depression, and MTLE + interictal psychosis derived from epilepsy surgery and control necropsies were investigated for neuronal density, granular layer dispersion, and MAP2 and tau immunohistochemistry. *Results*. Altered MAP2 and tau expression in MTLE and decreased tau expression in MTLE with psychosis were found. Granular layer dispersion correlated inversely with verbal memory scores, and with MAP2 and tau expression in the entorhinal cortex. Patients taking fluoxetine showed increased neuronal density in the granular layer and those taking haloperidol decreased neuronal density in CA3 and subiculum. *Conclusions*. Our results indicate relations between MAPs, granular layer dispersion, and memory that have not been previously investigated. Differential MAPs expression in human MTLE hippocampi with and without psychiatric comorbidities suggests that psychopathological states in MTLE rely on differential morphological and possibly neurochemical backgrounds. This clinical study was approved by our institution's Research Ethics Board (HC-FMRP no. 1270/2008) and is registered under the Brazilian National System of Information on Ethics in Human Research (SISNEP) no. 0423.0.004.000-07.

## 1. Introduction

Mesial temporal lobe epilepsy (MTLE) is the most common cause of intractable epilepsy in adults and is characterized by hippocampal sclerosis, neuronal loss, gliosis, dendritic alterations, mossy fiber sprouting, and granular layer dispersion [1–8]. Psychiatric comorbidities are frequent in MTLE patients, but their biological substrate is unknown [9, 10]. We have recently shown increased mossy fiber sprouting in patients with epilepsy and history of major depression and decreased in epilepsy with interictal psychosis, which may indicate a morphological basis for psychiatric symptoms in MTLE [11].

 Mossy fiber sprouting formation relies largely on proper arrangement of cytoskeletal components, such as microtubule-associated proteins (MAPs). Two of these major MAPs are MAP2 and tau. In animal models, increases in MAP2 and tau expression occur in the same timeframe of physiological and aberrant mossy fiber progression [12–14]. MAP2 is abundant in dendrites and neuron bodies, while tau is found in axons and neuron bodies [15, 16]. Whereas controversial findings have been reported on MAPs expression in schizophrenia and mood disorders [17–23], increased expression is found in the chronic phase of different animal models of epilepsy [12–14]. In human MTLE, only the hilar region has been examined in detail, showing no differences in MAP2 or tau expression in comparison to nonepileptic hippocampi [24].

 Therefore, we hypothesize that MAP2 and tau would also show differential expression in the hippocampal formation of MTLE patients with major depression and interictal psychosis. In addition, we investigated the possible correlation between MAP2, tau expression, granular layer dispersion, and further clinical characteristics.

## 2. Methods

### 2.1. Patients

We analyzed the hippocampal formation from MTLE specimens freshly collected in the operating room and nonepileptic controls from necropsy, collected between 4 and 7 hours after death. Tissue collection and processing were conducted according to the protocol approved by our institution's Research Ethics Board (HC-FMRP no. 1270/2008) and is registered under the Brazilian National System of Information on Ethics in Human Research (SISNEP) no. 0423.0.004.000-07.

 MTLE specimens were derived from 47 MTLE patients who underwent a standard en bloc anterior temporal resection (including 3-4 cm of the hippocampus) for medically intractable seizures. All had clinical neuropathological confirmation of HS, and in our selection we gave priority to the specimens exhibiting the whole hippocampal formation (hippocampus proper, subicular complex, and at least part of the entorhinal cortex). They were divided into 3 groups: 17 MTLE patients without any history of psychiatric disorder (MTLE group); 13 MTLE patients with interictal psychosis (MTLE + P group); and 17 MTLE patients with a diagnosis of major depression (MTLE + D group). The selected MTLE specimens were collected from 1998 to 2006. In these 8 years, 513 patients with temporal lobe epilepsy were operated in our center. According to a recent study comprising 10 years (1995–2005) of monitoring in our center, 9.5% of MTLE patients had comorbid psychotic disorders and 21% had mood disorders [25]. Considering that interictal psychosis corresponds to about 30% of psychosis of epilepsy [26], our MTLE + P group comprises almost the totality of interictal psychosis cases in the period. For comparison purposes, ten human nonepileptic control hippocampi from necropsies were processed and analyzed in the same manner as the surgical cases. Underlying diseases causing death were cardiomyopathy, pulmonary infarct or renal-hepatic failure, with no history of hypoxic episodes during agony, seizures, or neurological and psychiatric diseases. Furthermore, there was no evidence of brain pathological abnormalities on clinical postmortem examination of the mesial temporal structures. No correlation was seen between age of controls, postmortem time, MAPs expression, or neuronal density. A summary of clinical characteristics of all groups is depicted in Table 1.

### 2.2. Clinical Features of MTLE Patients

 All patients were referred for presurgical assessment due to drug-resistant seizures as defined in the literature [27]. Patients were evaluated at the Ribeirao Preto Epilepsy Surgery Program using standardized protocols approved by the institution's Ethics Committee and a written consent form was obtained from each patient. Presurgical investigation at the Epilepsy Monitoring Unit included detailed clinical history, neurological examination, interictal and ictal scalp/sphenoidal electroencephalography (EEG), neuropsychology evaluation, and intracarotid amobarbital memory and language procedure whenever deemed clinically necessary. 

 The definition of MTLE followed Engel's criteria [28]: (I) seizure semiology consistent with MTLE, usually with epigastric/autonomic/psychic auras, followed by complex partial seizures; (II) presurgical investigation confirming seizure onset zone in the temporal lobe; (III) anterior and mesial temporal interictal spikes on EEG; (IV) no lesions other than uni- or bilateral hippocampal atrophy on high resolution magnetic resonance imaging scans (reduced hippocampal dimensions and increased T2 signal); (V) clinical histopathological examination compatible with HS; (VI) no evidence of dual pathology identifiable by any of the assessment methods described (clinical, electrophysiology, neuroimaging, and histopathology). Exclusion criteria were: (I) focal neurological abnormalities on physical examination; (II) generalized or extratemporal EEG spikes; (III) marked cognitive impairment indicating dysfunction beyond the temporal regions.

 Information regarding antecedent of an initial precipitant injury, febrile seizures, seizure types, drug regimen, and estimated monthly frequency (within the two years prior to surgery) was retrospectively collected from medical records for each patient. Psychiatric evaluations were conducted in all MTLE patients. Each diagnosis of major depression was independently established during the presurgical evaluation by two psychiatrists with experience in psychiatric disorders associated with epilepsy, using the guidelines of the Diagnostic and Statistical Manual of Mental Disorders, 4th edition. Once a consensus on the classification of psychotic syndromes associated with epilepsy is lacking, and neither DSM-IV nor ICD-10 has addressed this issue specifically, the diagnosis of psychosis associated with MTLE was established according to Sachdev [29], meaning that patients with interictal psychosis did not experience the following: psychotic disorder temporally associated with seizures, changes in antiepileptic medications, epileptic status, delirium, and psychosis for paradoxical normalization. This group was defined by a prolonged psychotic state that was not related to the epileptic seizures. Typically, the psychotic states closely resemble schizophrenia, with paranoid ideas which might become systematized, ideas of influence, and auditory hallucinations often of a menacing quality. The points of difference are common religious coloring of the paranoid ideas, tendency of the affect to remain warm and appropriate, and no typical deterioration to the hebephrenic state [30]. Patients had no history of previous psychiatric disorders (prior to seizure onset) or of substance dependence at any time. Global IQ was calculated after neuropsychological tests (complete WAIS-III or WAIS-R protocol). 

### 2.3. Tissue Collection and Immunohistochemical Processing

 Specimens were segmented into 1 cm blocks transversely oriented to the hippocampal long axis. Blocks were placed in buffered paraformaldehyde (Sigma, St. Louis, MO, USA). After 48–96 hours, specimens were paraffin embedded for immunohistochemistry.

 Immunohistochemistry was performed with antibodies that identified immunoreactivity for Neu-N, a nuclear protein found in the nuclei of mature neurons (1 : 1000 dilution; Chemicon-Millipore, Billerica, MA, USA), and for the MAPs: total MAP2 (independent of phosphorylation state, H-300, 1 : 200 dilution; Santa Cruz Biotechnology, Santa Cruz, CA, USA), and total tau (independent of phosphorylation state, H-150, 1 : 100 dilution; Santa Cruz Biotechnology, Santa Cruz, CA, USA). According to the manufacturers and selected references [31–34], these antibodies show no cross-reactivity with other MAPs and recognize only its own isoform either as a single band or as a doublet. Briefly, paraffin-embedded MTLE and control hippocampi were processed together for each antibody as described in Kandratavicius et al.'s work [11], with overnight incubation at room temperature and developed simultaneously for 10 min in 0.05% 3, 3′-diaminobenzidine tetrahydrochloride (Pierce, Rockford, USA) and 0.01% hydrogen peroxide (Merck, Darmstadt, Germany). After sufficient colorization, the reaction was halted by washing in several rinses of distilled water, dehydrated through graded ethanol to xylene (Merck, Darmstadt, Germany), and cover-slipped with Krystalon (EM Science, Gibbstown, NJ, USA). Adjacent sections were hematoxylin-eosin stained (Laborclin, Pinhais, Brazil) and examined for tissue integrity. Control sections without the primary antisera did not reveal staining (data not shown).

### 2.4. Cell Count and Semiquantitative Analysis of Immunohistochemistry

 MTLE and control hippocampi were compared for neuronal density using Lorente de No's classification [35], which included dentate gyrus' granular and subgranular cells, polymorphic hilar neurons (limited to a region between stratum granulosum and CA4 pyramidal cells, being at least 50 *μ*m from the stratum granulosum and 100 *μ*m from CA4), as well as pyramidal cells in CA4 (the portion of Ammon's horn that permeates the inner part of the dentate gyrus), CA3, CA2, CA1, prosubiculum, subiculum, parasubiculum, and entorhinal cortex layer III. Cell densities (neurons per cubic millimeter) were estimated in 8 *μ*m Neu-N-stained slices at 400x magnification with a morphometric grid methodology using Abercrombie's correction [36] as previously described and well established in the literature for surgical hippocampal fragments [1, 3, 8, 11, 37–41]. We also performed measurements of granular layer dispersion using Neu-N-stained sections and the straight line tool of Image J analysis system (NIH, USA, public domain). The inferior limit was considered as the furthest granular neuron in the subgranular zone and the superior limit the furthest granular neuron invading the molecular layer.

 Images were collected and digitized with a high-resolution CCD monochrome camera (model C2400-75H, Hamamatsu Photonics K.K., Japan) attached to a BX60F5 microscope (Olympus Optical Co., Japan). This method was used to obtain digitized images of Neu-N-, MAP2-, and tau-stained slides. Uniform luminance was maintained and checked every 10 measurements using an optical density standard and a gray value scale ranging from 0 (white) to 255 (black).

 Adjacent slides to those examined for neuronal density were analyzed for MAPs expression. In brief, all digitized images were analyzed with Image J software, following the same criteria: (I) the software identifies the gray value distribution of a subfield's digital image (total area for each subfield = 313.7 *μ*m × 235.3 *μ*m); (II) the immunoreactive area is selected (i.e., positive stained pixels), limited to a threshold range; and (III) the threshold range is presettled based on control group sections to exclude the low-intensity gray value of background staining from the analysis. A similar approach was used by our group elsewhere [42]. Results for granular layer included granular cell layer per se and proximal molecular layer. Analyses were conducted by one investigator (L.K.), blind to group classification.

### 2.5. Data Analysis

 Data were analyzed using the statistical program PAWS (version 18.0) and SigmaPlot (version 11.0). Groups were compared using analysis of variance (ANOVA one-way analysis with Bonferroni post hoc test) or unpaired *t*-test for variables with normal distribution and Kruskal-Wallis one-way analysis of variance on ranks (with Dunn post hoc test) or Mann-Whitney rank sum test for variables without normal distribution. Fisher's exact test was applied for comparison of relative frequencies of clinical variables between groups. Other statistical tests included Pearson correlation analyses. Statistical significance was set at *P* < 0.05 and values presented as mean ± SD.

## 3. Results

### 3.1. Clinical Profiles

 The four patients groups did not show significant differences in gender, age, or collected side (Table 1). Clinical variables such as presence of an initial precipitant injury, age of first seizure and seizure onset, seizure frequency and epilepsy duration, HS side, handedness, IQ, years at school, and performance in verbal memory tests were homogeneously distributed among MTLE groups. An increased proportion of MTLE + P patients exhibited worse performance in nonverbal memory tasks when compared to MTLE patients without psychiatric comorbidities (Fisher's exact test, *P* = 0.03).

 All epileptic patients were on antiepileptic drugs (carbamazepine, oxcarbazepine, phenobarbital, and/or phenytoin). In addition, patients were also taking benzodiazepines (MTLE group: 14 of 17; MTLE + D: 14 of 17; MTLE + P group 8 of 13), fluoxetine (MTLE + D: 7 of 17), and haloperidol (MTLE + P group: 8 of 13). No differences in neuropsychological tests between patients taking or not taking benzodiazepines, fluoxetine, or haloperidol were seen. Possible influence of fluoxetine and haloperidol on neuronal density and MAPs expression will be explored in the next sections.

### 3.2. Neuropathological Characterization: Neuronal Density

**Figure 1:**
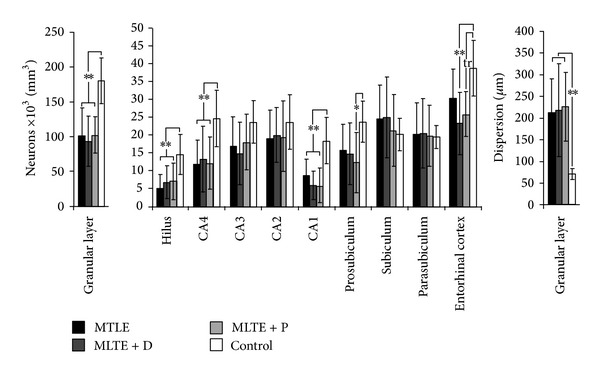
Neuronal density in human hippocampal formation subfields. Neuronal density values from MTLE (black bars), MTLE + D (gray bars), MTLE + P (light gray bars), and from nonepileptic controls (white bars) are indicated as mean ± std. deviation. Asterisks indicate significant statistical difference (double: *P* < 0.01, single: *P* < 0.05) between epileptics and control group. Neuronal loss was observed in granular layer, hilus, CA4, CA1, prosubiculum, and entorhinal cortex. A statistical trend (tr: 0.05 ≥ *P* ≤ 0.07) to decreased neuronal density in MTLE + P entorhinal cortex when compared to control can also be seen. Dispersion in the granular layer was greater in epileptic patients than in controls.

 Evaluation of epileptogenic and control hippocampal formation showed reduced neuron density in the granular layer, hilus, CA4, and CA1 of all MTLE groups when compared to control (Figure 1, asterisks). We also found neuron density reduction in the entorhinal cortex of MTLE patients with major depression and a trend in the interictal psychosis group when compared to controls. In addition, decreased neuronal density was found in prosubiculum of MTLE + P specimens. MTLE groups exhibited increased granular layer width when compared to controls (ANOVA *F* (3, 53) = 7.04, *P* = 0.001), with no statistically significant differences among MTLE groups (ANOVA *F* (2, 44) = 0.01, *P* = 0.98).

 Interestingly, length of dispersion in the granular layer correlated inversely with scores in neuropsychological verbal tests (*R* = −0.47, *P* = 0.02), similar to what we found in a different series (unpublished data). Performances in verbal memory tests were similar among epileptic groups, and psychiatric diagnosis was not a factor of influence. When patients were all grouped according to their verbal scores, we saw that performance below average was indeed related to increased granular cell layer dispersion (below average: 263.5 ± 95.2 *μ*m  versus average and above: 156.4 ± 63.5 *μ*m, *t* (43) = 3.15, *P* = 0.005). Also, MTLE + D patients taking fluoxetine presented with increased neuronal density in the granular layer when compared to those not taking fluoxetine (114.5 ± 37.6 × 10^3^ neurons/mm^3^  versus 74.8 ± 27.0 × 10^3^ neurons/mm^3^, *t* (15) = 2.37, *P* = 0.03). In contrast, MTLE + P patients taking haloperidol showed decreased neuronal density when compared to those not taking haloperidol in CA3 (12.7 ± 7.4 × 10^3^ neurons/mm^3^ versus 25.0 ± 1.7 × 10^3^ neurons/mm^3^, *t* (8) = −2.74, *P* = 0.04) and subiculum (17.4 ± 9.4 × 10^3^ neurons/mm^3^ versus 31.6 ± 5.1 × 10^3^ neurons/mm^3^, *t* (11) = −3.20, *P* = 0.01).

### 3.3. MAPs Expression

**Figure 2:**
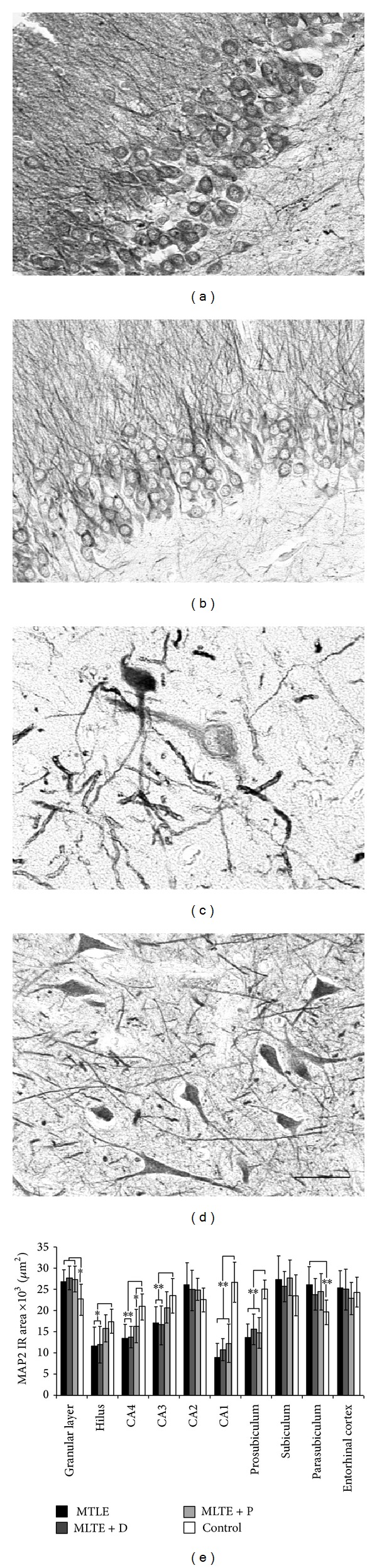
MAP2 expression in MTLE specimens with and without psychiatric comorbidities and in nonepileptic controls. MTLE granular layer (a) exhibited increased MAP2 expression when compared to control group (b). In the hilus, decreased expression can be seen in hyperthrophic hilar neurons (c) while in controls neurons exhibited an elongated profile and a defined dendritic mesh (d). We found that MAP2 immunoreactive (IR) area values (e) decreased in the hilus, CA4, CA3, CA1, and prosubiculum subfields of MTLE hippocampus and increased in MTLE granular layer and parasubiculum. Asterisks indicate significant statistical difference (double: *P* < 0.01, single: *P* < 0.05) between epileptics and control group. Values from MTLE (black bars), MTLE + D (gray bars), MTLE + P (light gray bars), and from nonepileptic controls (white bars) are indicated as mean ± std. deviation. Bar (a–d): 50 *μ*m.

 Distinct MAP2 expression was seen in neuronal bodies and dendrites (Figures 2(a)–2(d)). Hippocampal formation subfields of epileptic patients with increased MAP2 expression when compared to controls were the granular layer and parasubiculum and decreased in the hilus, CA4, CA3, CA1, and prosubiculum (Figure 2(e), asteriks). Of note, MAP2 expression correlated inversely with neuron density in CA1 (a trend, *R* = −0.30, *P* = 0.07), prosubiculum (*R* = −0.52, *P* = 0.002), and subiculum (*R* = −0.38, *P* = 0.01), suggesting that neurons in CA1, prosubiculum, and subiculum may exhibit some degree of dendritic remodeling. Also, decreased hilar (*R* = −0.46, *P* = 0.03) and entorhinal cortex (*R* = −0.65, *P* = 0.004) MAP2 expression was related to increased granular cell layer dispersion. No differences in MAP2 expression were seen among epileptic groups. In the hilus and CA3, MTLE + P specimens exhibited the same amount of MAP2 immunoreactive area than controls, while in the other two epileptic groups MAP2 expression was decreased. MTLE + D patients taking fluoxetine showed increased MAP2 expression in CA2 (30158.0 ± 1150.5 *μ*m^2^ versus 20967.8 ± 2006.3 *μ*m^2^, *t* (14) = 9.15, *P* < 0.0001).

**Figure 3:**
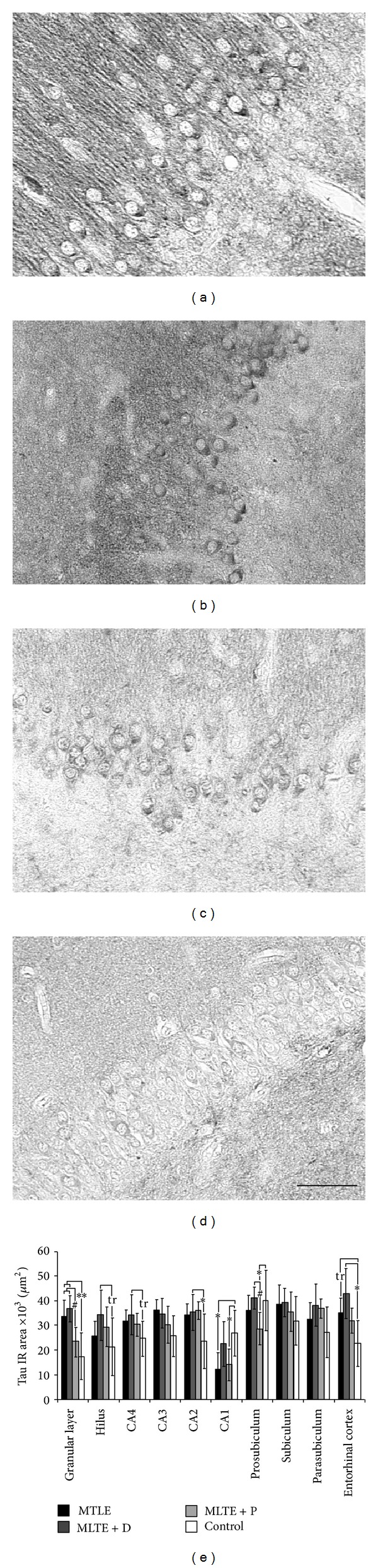
Tau expression in MTLE specimens with and without psychiatric comorbidities and in nonepileptic controls. MTLE (a) and MTLE + D (b) exhibited increased tau expression in granular layer when compared to MTLE + P (c) and control group (d). Immunostaining was enriched in cell bodies, molecular layer, and surrounding neuropil. We found that tau immunoreactive area values (e) increased in most subfields of epileptic patients when compared to controls (double asterisk: *P* < 0.01, single asterisk: *P* < 0.05). A statistical trend (tr: 0.05 ≥ *P* ≤ 0.07) to increased tau expression in the hilus, CA4, and entorhinal cortex can also be seen. Regions with differences among epileptic groups were the granular layer and prosubiculum, where MTLE + P patients presented with decreased tau expression (hash sign, *P* < 0.05). Values from MTLE (black bars), MTLE + D (gray bars), MTLE + P (light gray bars), and from nonepileptic controls (white bars) are indicated as mean ± std. deviation. Bar (a–d): 50 *μ*m.

 Tau expression was evident in neuronal cell bodies and axons. In the granular layer (Figures 3(a)–3(d)), differential tau expression can be seen among the groups, closely resembling the profile of our previous findings of differential aberrant sprouting of mossy fibers, the granular cells' axons [11]. Quantification of tau immunoreactive area revealed marked differences between epileptogenic specimens and controls (Figure 3(e), asterisks) and among epileptic groups (Figure 3(e), hash signs in the granular layer and prosubiculum). Patients with interictal psychosis exhibited lower tau values in the granular layer, CA1, and prosubiculum, while patients with major depression presented with increased tau values in most hippocampal subfields. When patients were grouped according to their verbal scores, we saw that performance below average was related to increased tau expression in CA2 (below average: 36530.5 ± 5362.3 *μ*m^2^ versus average and above: 28247.9 ± 2894.2 *μ*m^2^, *t* (41) = 2.25, *P* = 0.02). Interestingly, increased tau expression in CA2 was directly correlated with epilepsy duration (*R* = 0.56, *P* = 0.02), suggesting a progressive course of tau accumulation related to the epileptogenic process. By contrast, tau expression in the entorhinal cortex was inversely correlated with granular cell layer dispersion (*R* = −0.74, *P* = 0.02). MTLE + D patients taking fluoxetine showed decreased tau expression in the entorhinal cortex (35948.2 ± 7466.1 *μ*m^2^ versus 52297.0 ± 6991.3 *μ*m^2^, *t* (9) = −2.94, *P* = 0.03). Regarding MTLE + P patients taking haloperidol, no differences in MAP2 or tau expression were seen when compared to those not taking haloperidol.

 All epileptic patients were taking antiepileptic drugs (AEDs) either as monotherapy or polytherapy at time of surgery (mainly carbamazepine, followed by phenytoin and phenobarbital). No differences in those taking AEDs were seen among epileptic groups (Chi-square, *P* > 0.87). No patient was taking topiramate. There were no differences in averaged MAPs expression or neuronal densities between patients taking a given AED as compared to those not taking that drug (unpaired *t*-tests, 0.60 > *P* > 0.93).

## 4. Discussion

 Although studies with hippocampi from MTLE patients have been done for more than a century (for review, see [43]), and several abnormalities in the hippocampal formation of patients with schizophrenia and major depression have also been described (for review, see [9, 10]), few neuropathological studies are available to date regarding the strong association between epilepsy and psychiatric comorbidities. We have previously reported in another series of patients differential mossy fiber sprouting in MTLE patients with psychosis and with major depression [11]; this result is in agreement with our present finding of tau expression in the granular layer.

 Since the description of granular cell layer dispersion in epileptic patients by Houser [6], other studies have provided some insight into possible mechanisms such as neo-migration related to gliogenesis, mossy fiber sprouting and extracellular matrix disturbances [44–46]. More recently, pieces of evidence point to granular cell dendritic alterations related to dispersion [47, 48]. In our series, we found correlation between increased granular layer width and decreased hilar MAP2 expression, suggesting that loss of original hilar targets may also contribute to dispersion. The entorhinal cortex is one of the major sites that projects to the dentate gyrus [49], and decreased MAP2 and tau expression in the entorhinal cortex is likely a sign of missing local dendrites and axons that would lead to impaired connectivity between the granular layer and extrahippocampal sites. Intact communication between the hippocampus and surrounding limbic structures is vital to the expression of behavior and cognition [9], and may also have an important role in granular layer dispersion, which was not previously investigated. Another significant finding was the relation between increased granular layer dispersion and decreased scores in verbal memory tests. The nature of this particular association is still unclear, but we can speculate that granular layer dispersion contributes in a negative way to the altered circuitry of epileptogenic hippocampal formation. In fact, granular cell loss and dispersion have been associated with poor memory performance in MTLE patients [50, 51]. Moreover, patients taking fluoxetine showed increased neuronal density in the granular layer when compared to those not taking fluoxetine. Increased hippocampal neurogenesis after chronic fluoxetine treatment has been demonstrated in rats and nonhuman primates [52, 53], and it is probably related to its therapeutic effects [54]. Decreased neuronal density seen in patients taking haloperidol is in agreement with pieces of evidence that antipsychotics may reduce brain tissue volume [55].

 In the present series, we found better preservation of dentritic/MAP2-related elements in the hilus and CA3 of MTLE patients with psychosis. Of note, similar regions have been described as MAP2 enriched in the hippocampus of schizophrenic patients [18]. We hypothesize that an increased MAP2 immunoreactive dendritic arborisation especially in the hilus and CA3 of MTLE patients with psychosis could be related to our previous results of decreased mossy fiber sprouting in MTLE + P patients [11]. One of the theories that may explain aberrant mossy fiber sprouting in epilepsy regards loss of the physiological mossy fibers original targets, that is, majorly, hilar, and CA3 cells [39, 56, 57]. If their original contacts are less damaged as suggested by a preserved MAP2 immunoreactivity, we could expect less aberrant sprouting, as we have documented. In addition, we found that MTLE + D patients taking fluoxetine showed increased MAP2 expression in CA2, in agreement with studies in animal models of depression showing hippocampal upregulation of dendritic-related proteins after chronic fluoxetine administration, which was also related to improvement in behavior and memory [58–60].

 Tau expression has been scarcely examined in MTLE and schizophrenia, and no significant differences were found between patients and controls [24, 61]. Here, we analyzed the whole hippocampal formation for the first time and reported an increased tau expression in most hippocampal subfields of epileptic patients, as well as decreased expression in the granular layer and in CA1 and prosubiculum of MTLE + P patients. Interestingly, expression levels of other axonal proteins such as growth-associated protein 43, synaptophysin, vesicular glutamate transporters, reelin, and complexins are also decreased in the hippocampus and prefrontal cortex of schizophrenic patients [62–65]. Together, these studies show that despite neo-synaptogenesis inherent to some subfields of the MTLE hippocampus [66], synaptic pathology as seen in schizophrenia might disrupt this scenario in MTLE + P patients. In fact, similarities between schizophrenia and interictal psychosis have also been detected in hippocampal homogenates, such as increased activity of phospholipase A2 [67]. In addition, we found that increased tau expression in CA2 was related to poor verbal memory performance, similar to the results of tau overexpression in animal models [68, 69], indicating that normal learning function requires a fine balance between stability and instability in microtubules [70]. Another finding was decreased tau expression in the entorhinal cortex of MTLE + D patients taking fluoxetine, in agreement with tau mRNA downregulation described in Parkinson's patients taking fluoxetine [71]. Positive effects of fluoxetine treatment have also been documented in Alzheimer's disease [72].

 Inherent to our findings and conclusions, it is important to acknowledge some limitations of our study. Even with a relatively small sample size, the present results suggest that further studies exploring MTLE and related comorbidities are worthwhile. In fact, since all MTLE surgical specimens were freshly collected and submitted to identical processing, differences among them are particularly relevant. Also, we used as controls hippocampi from necropsy with longer delays before fixation than those for our surgical specimens. There is evidence that postmortem intervals up to 24 h do not interfere significantly with protein levels, cell morphology, and tissue integrity [42, 73, 74]. Furthermore, our controls showed no correlations between postmortem delays and MAPs expression. Hence, it seems reasonable to compare surgically collected tissue to autopsies as has been suggested by other authors [8, 38, 40, 42, 66].

 In conclusion, we have examined the expression patterns of MAP2 and tau in the hippocampal formation subfields of MTLE patients with and without psychiatric comorbidities and have correlated them with features indicative of cognitive impairment. Our results indicate that structural proteins are central components of rewiring mechanisms that may underlie pathophysiological states. Altered MAPs expression and related granular layer dispersion in the hippocampus of MTLE may partially explain the impaired memory commonly seen in these patients. Our results are consistent with our previous series and indicate that psychopathological states in MTLE rely on differential morphological and possibly neurochemical backgrounds. Future studies with human tissue and animal models will be needed to determine the origin and pathogenic significance of synaptic/dendritic pathology in MTLE with psychiatric comorbidities.

## Figures and Tables

**Table 1:** Demographic and clinical data.

	MTLE	MTLE + D	MTLE + P	Controls	Statistics
Male (*n*)	9	7	7	5	No difference
Female (*n*)	8	10	6	5	

IPI present (*n*)	9	7	8	n.a.	No difference
IPI absent (*n*)	8	10	5	n.a.	

Age of first seizure (years)	4.2 ± 3.8	6.0 ± 6.4	6.5 ± 8.4	n.a.	No difference

Age when seizures became recurrent or age of onset (years)	10.3 ± 6.4	12.8 ± 7.9	14.6 ± 10.0	n.a.	No difference

Seizure type: CPS (*n*)	8	3	4	n.a.	No difference
Seizure type: SGS (*n*)	9	14	9	n.a.	

Seizure frequency (monthly)	10.8 ± 10.0	15.2 ± 10.7	8.0 ± 5.4	n.a.	No difference

Right HS (*n*)	8	8	6	n.a.	No difference
Left HS (*n*)	8	7	5	n.a.	
Bilateral HS (*n*)	1	2	2	n.a.	

Right handedness (*n*)	15	14	13	n.a.	No difference
Left handedness (*n*)	1	2	0	n.a.	
Bilateral handedness (*n*)	1	1	0	n.a.	

Memory in verbal tasks: average or above (*n*)	5	7	2	n.a.	No difference
Memory in verbal tasks: below average (*n*)	12	10	11	n.a.	

Memory in nonverbal tasks: average or above (*n*)	11	8	3	n.a.	Fisher's exact test, *P* = 0.03 (MTLE × MTLE + P)
Memory in nonverbal tasks: below average (*n*)	6	9	10	n.a.	

Full-scale IQ	85.7 ± 11.9	85.8 ± 8.1	77.8 ± 8.2	n.a.	No difference

Years at school	6.8 ± 3.2	6.8 ± 4.7	3.9 ± 2.5	n.a.	No difference

Age at surgery (or at death for controls, in years)	37.1 ± 6.3	37.3 ± 5.2	38.8 ± 4.3	47.3 ± 19.1	No difference

Duration of epilepsy (years)	26.7 ± 7.6	24.5 ± 9.1	24.2 ± 11.4	n.a.	No difference

Collected side: right (*n*)	9	9	7	5	No difference
Collected side: left (*n*)	8	8	6	5	

Values indicated as mean ± std. deviation when applicable. IPI: initial precipitant injury; CPS: complex partial seizure; SGS: secondarily generalized seizures; HS: hippocampal sclerosis; n.a.: not applicable.
